# The therapeutic implications of the genomic analysis of malignant pleural mesothelioma

**DOI:** 10.1038/s41467-021-22142-y

**Published:** 2021-03-22

**Authors:** Marjorie G. Zauderer

**Affiliations:** MSK Mesothelioma Program, Thoracic Oncology Service, Department of Medicine. Memorial Sloan Kettering Cancer Center, New York, NY USA

**Keywords:** Cancer genomics, Mesothelioma

## Abstract

Delineation of the genomic complexities of malignant pleural mesothelioma (MPM) has lagged behind other malignancies. Zhang et al. meaningfully add to our understanding of MPM, and their findings emphasize the need to combine drug development efforts with appropriate predictive biomarkers.

## Historical beliefs about mesothelioma

For far too long, malignant pleural mesothelioma (MPM), a cancer of the mesothelial cells lining the pleural cavity, has been approached as a single homogeneous disease entity. Our relatively basic classification based on histology^1^ helps us prognosticate but does not meaningfully inform management decisions, except perhaps for surgery in sarcomatoid disease^2^. Because of a variety of beliefs surrounding the etiology, prognosis, and treatment of MPM, efforts to delineate the genomic complexities of this disease have lagged behind those of other malignancies. With rational drug development successes in other common malignancies, such as lung cancer, as well as the advent of increasingly powerful next-generation sequencing and analytic tools, we are beginning to identify and define the complex genomic landscape of MPM so personalized therapies can be developed.

## The genomics of MPM

A decade ago, Dr. Ladanyi and colleagues reported the first integrated genomic analysis of MPM^3^. They examined 25 potential driver genes and found a high rate of non-synonymous mutation in *BAP1*, confirmed frequent inactivating mutations in *NF2*, and described missense mutations in *LATS1* and *LATS2*. Subsequently, in 2016, Dr. Bueno and colleagues analyzed RNA sequencing data from 216 MPM tumors which confirmed and expanded prior findings^4^. In addition to common alterations in *BAP1* and *NF2*, alterations were identified in *TP53*, *SETD2*, *DDX3X*, *ULK2*, *RYR2*, *CFAP45*, *SETDB1*, and *DDX51*. Based on the robust genomic heterogeneity observed in MPM, Bueno et al. proposed four distinct molecular disease subtypes: sarcomatoid, epithelioid, biphasic-epithelioid, and biphasic-sarcomatoid. The Cancer Genome Atlas’ (TCGA) comprehensive integrated genomic study of MPM, reported by Dr. Hmeljak and colleagues in 2018, identified prognostic molecular subsets independent of histology^5^, and also defined a novel subtype with extensive loss of heterozygosity and mutations in *TP53* and *SETDB1*. Taken together, these studies paint a picture of the common recurring alterations in mesothelioma and begin to define disease subsets based on genomic characteristics.

Multiple novel targets and pathways of interest have been identified from genomic studies of MPM. Unlike many malignancies with mutations in growth-regulating kinases, the genomic landscape of MPM is primarily characterized by alterations in tumor suppressor genes. Unfortunately, therapeutic targeting of these tumor suppressor alterations remains elusive. Furthermore, no predictive subsets or markers have been identified to help with patient selection for various treatments, and intra-tumoral heterogeneity has not been evaluated. It is in this context that Zhang et al. present a body of work examining clonal architecture and its influence on the tumor microenvironment in MPM^6^.

## Modeling MPM

Zhang et al. describe their creation of a platform, entitled MEDUSA (Mesothelioma Evolution: Deciphering Drugable Somatic Alterations), used to infer a model of MPM tumorigenesis^6^. Through multi-regional exome sequencing of 90 tumor samples collected at the time of extended pleurectomy/decortication from anatomically stereotyped regions in 22 patients with MPM, and with whole blood germline controls, Zhang and colleagues created phylogenetic tree topology in order to identify potential evolutionary constraints that could be exploited for drug development. Extensive inter-patient and intra-tumor heterogeneity was identified.

Linear evolutionary trees, comprised of monophyletic clones arising from a common node, modeled 64% of the cases. Branched trees, comprised of polyphyletic clones arising from subclonal nodes, described the remaining 36% of cases. The evolutionary analysis suggested that *BAP1* events occur early in the evolution of MPM as evidenced by their presence in almost all subclones. By contrast, *NF2* events occur late and are therefore only evident in some branches. Clonal positive selection was demonstrated for *NF2*, *BAP1*, *SETD2*, *FBXW7*, and *PRELID1*. Subclonal selection was only identified for *NF2*. No whole-genome haploidization or whole-genome doubling events were noted. Twenty-four percent of samples had copy number alterations with losses more significant than gains, and only 5% of these were subclonal. There was also some evidence of allelic heterogeneity. Homozygous deletion of *CDKN2A/B* and *MTAP* occurred only as clonal events. Detectable circulating free DNA was associated with inferior survival. Taken together, these findings demonstrate that in addition to substantial intra- and inter-tumoral heterogeneity, there are common key genomic events for clonal and subclonal evolution which lend themselves to focused drug development.

Zhang et al. also explored how intra-tumoral heterogeneity modulates host immune surveillance or immune escape. High clonal copy number burden was associated with greater systemic inflammation as measured by neutrophil:lymphocyte and platelet:lymphocyte ratios. Additionally, the most highly branched tumors had higher T-cell infiltration. Highly branched tumors also had higher neoantigen burden which is associated with immunoediting via HLA loss of heterozygosity and can lead to immune escape. These data are compelling evidence that clonal architecture modulates immune surveillance and is a provocative potential mechanism of resistance to immune checkpoint inhibitor therapy in MPM.

## Moving beyond the limited therapeutic options in MPM

**Fig. 1:**
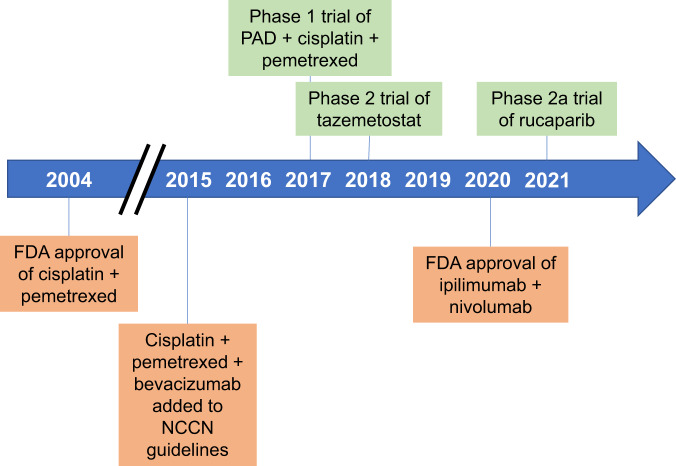
Timeline of drug approvals/recommendations and personalized clinical trials in malignant mesothelioma. Genome-informed clinical trials are shown above the timeline in green. Drug approvals and recommendations are shown below the timeline in orange. FDA U.S. Food and Drug Administration, NCCN National Comprehensive Cancer Network, PAD pegylated arginine deaminase.

Approved therapeutic options in MPM had remained largely unchanged for the last 16 years (Fig. 1). This study by Zhang et al. comes at an opportune time as the treatment approach in MPM is now evolving. Based on the positive results of the Checkmate-743 trial^7^, the combination of ipilimumab and nivolumab has received FDA approval and is included in the NCCN guidelines as a first-line treatment option for patients with MPM. While this is an important advance for the treatment of MPM, many questions remain unanswered. Responses differ by histology and PD-L1, but no compelling predictive marker to facilitate treatment selection has been identified. This underscores the importance of identifying reliable predictive biomarkers based on the underlying biologic mechanisms of response and resistance for all treatment options.

Future clinical trials need to focus on predictive biomarkers for novel therapies. In order to meaningfully advance outcomes in this disease, we must develop effective therapies and identify the patients most and least likely to benefit from them. Exceptional responders were identified in many early phase clinical trials in MPM, but without a biomarker, these compounds were abandoned for lack of efficacy. In other cancers, biomarkers have been identified that predict a patient’s response to drugs; for instance, larotrectinib for TRK fusion-positive cancers^8^. A parallel approach in MPM is possible, but only with more studies like those described here by Zhang et al.^6^.

The tremendous diversity of MPM described by Zhang et al. and others means that similar rational drug development and trial design is needed for this disease. To do this, we must abandon the long-held, mistaken assumptions of homogeneity within and between MPM tumors as well as the nihilism around conducting MPM trials in selected populations enriched for response. There have been some efforts in this space to establish the feasibility of selecting patients based on histology^9^ or BAP1 loss^10,11^. Now, we need more biologic inquiry to fuel the next generation of potential therapies and their target populations. This body of work from Zhang et al. is an important step toward those goals with its characterization of the extensive exomic variation within patients that has long been overlooked due to the low metastatic potential of MPM. By building on these discoveries and the relationships identified between intratumoral heterogeneity and immune surveillance, we can better identify potentially druggable alterations and create personalized therapies for patients with MPM. Work like this hails the dawn of a new age for drug discovery and development in MPM, and I am optimistic for what lies ahead.
